# Effects of dry needling on vertical jump performance in female volleyball players. A randomized controlled trial

**DOI:** 10.3389/fspor.2024.1470057

**Published:** 2024-09-04

**Authors:** Arturo Ladriñán-Maestro, Jorge Sánchez-Infante, Daniel Martín-Vera, Jose Angel Del-Blanco-Múñiz, Diego Domínguez-Balmaseda, María José Guzmán-Pavón, Alberto Sánchez-Sierra

**Affiliations:** ^1^ School for Doctoral Studies and Research, Universidad Europea de Madrid, Madrid, Spain; ^2^ Research Group on Exercise Therapy and Functional Rehabilitation, Faculty of Sports Sciences, Universidad Europea de Madrid, Madrid, Spain; ^3^ Faculty of Physiotherapy and Nursing of Toledo, Universidad de Castilla-La Mancha, Toledo, Spain; ^4^ Faculty of Sports Sciences, Universidad Europea de Madrid, Madrid, Spain; ^5^ Physiotherapy Research Group of Toledo (GIFTO), Faculty of Physiotherapy and Nursing, Universidad de Castilla-La Mancha, Toledo, Spain; ^6^ Faculty of Health Sciences, Universidad Francisco de Vitoria, Madrid, Spain; ^7^ Clínica Axium Salud Funcional, Madrid, Spain; ^8^ Masmicrobiota Group, Faculty of Health Sciences, Universidad Europea de Madrid, Madrid, Spain; ^9^ Real Madrid Graduate School, Faculty of Sports Sciences, Universidad Europea de Madrid, Madrid, Spain; ^10^ ABC-age Research Group, Cuenca, Spain; ^11^ Clínica Sierra Varona SL, Toledo, Spain; ^12^ Department of Physiotherapy, Faculty of Health, Camilo José Cela University, Villanueva de la Cañada, Madrid, Spain; ^13^ Department of Physical Therapy, Faculty of Health Sciences, Universidad Alfonso X El Sabio, Madrid, Spain

**Keywords:** physical therapy, rehabilitation, sports medicine, sports performance, injury prevention

## Abstract

**Introduction:**

Injury prevention and performance enhancement are paramount goals in sports. Myofascial Pain Syndrome, primarily caused by myofascial trigger points, can result in referred pain, stiffness, muscle shortening, and weakness. This study aimed to assess the impact of dry needling (DN) on latent myofascial trigger points on vertical jump performance in female volleyball players.

**Methods:**

A single-blind, randomized controlled clinical trial was conducted with twenty-six healthy female volleyball players who had no lower limb injuries in the last six months, exhibited latent trigger points in the triceps surae muscles, and were familiar with the countermovement jump test. Participants were randomly assigned to either a control group or an experimental group (which received a single DN session). Vertical jump performance variables, including jump height as the primary outcome, were assessed using a force platform at five time points: before the intervention, immediately post-treatment, 24 h post-treatment, 72 h post-treatment, and one-week post-intervention.

**Results:**

The experimental group showed significantly lower values for vertical jump height, flight time, velocity, strength, and power immediately after the needling intervention (*p* < 0.05). However, these values were significantly higher one-week post-intervention across all variables (*p* < 0.01). These findings indicate that DN initially decreases jumping performance, but improvements are observed one week after the intervention. In the comparison between groups, the experimental group exhibited higher values at the one-week follow-up for vertical jump height, flight time, speed, and power compared to the control group (*p* < 0.05).

**Conclusions:**

DN appears to be an effective technique for improving vertical jump performance in female volleyball players one week after its application.

**Clinical Trial Registration:**

ClinicalTrials.gov, identifier (NCT06184672).

## 1 Introduction

At present, the implementation of injury prevention techniques has become indispensable for maintaining athletes in optimal health, enabling them to achieve superior performance (1). Myofascial Trigger Points (MTPs) serve as a primary clinical manifestation in Myofascial Pain Syndrome (MPS), contributing to localized muscle pain (2). MTPs are hyperirritable nodules characterized by focal pressure pain within tense, palpable bands of skeletal muscle. Upon palpation, they induce pain and elicit local spasms, further producing distant motor and autonomic effects (3). Key diagnostic characteristics of MTPs in MPS encompass palpable tension and a tensile band, distinguishing them from other trigger point types. Additional features include a local spasm response within the taut band, pain referred to other body regions, stiffness, shortening, muscle weakness, and pain during muscle contraction. Consideration must be given to both direct triggers (e.g., trauma, overloads, cooling) and indirect triggers (e.g., other MTPs, visceral disease, radiculopathy, joint dysfunction or inflammation, and stress) (4).

MTPs are subject to various classifications. In terms of clinical presentation, they can be categorized as active trigger points (AMTP) or latent trigger points (LMTP). While both may result in dysfunction, only AMTPs induce spontaneous pain in patients. Anatomically, they can be central (located in the central part of muscle fibers) or insertional (in the area of muscle fiber insertion). It is crucial to distinguish MTPs from trigger points in other tissues, such as skin, ligaments, or periosteum. The prevalence of LMTP in lower limb muscles, particularly in the gastrocnemius muscle, is noteworthy, especially in women (5). Conservative treatment approaches for managing MTPs involve manual and instrumental techniques, with the primary goal of normalizing the length of shortened sarcomeres within MTPs. Invasive treatments are also available and represent some of the most effective procedures (2). Various needling techniques for MTPs, collectively termed “dry needling (DN)”, have been described, with the Hong technique demonstrating superior effectiveness compared to superficial needling techniques (6). The available evidence on the effectiveness of DN in sports recovery is limited and contradictory. Some studies have found no improvements with DN treatment following exercise for delayed onset muscle soreness (7), while others have reported improvements in pain threshold under the same conditions (8).

There is evidence of a higher prevalence and incidence of LMTP in athletes compared to sedentary individuals (9, 10). As previously mentioned, these LMTP have a range of negative effects on athletic performance, including vertical jump performance. Notable among these effects are pain, alterations in muscle contraction, and increased fatigability of the affected musculature (11, 12).

Volleyball is a team sport that features rapid acceleration-deceleration movements, jumping, short-duration, high-intensity efforts, changes of direction, and ball-striking (13). One of the most common tests described in the literature to assess physical fitness in various populations is the vertical jump test (14) and its variations. Primarily utilized in sports, these tests evaluate muscle power in the lower limb (15) and find applications in diverse demographics, including children (16) and the elderly (17), with potential benefits for improving bone density (18). Vertical jump ability plays a fundamental role in volleyball performance, being closely related to both defensive and offensive aspects of the game, and serving as an important marker of players' competitive level (19, 20).

Currently, in the world of sports, injury prevention and recovery strategies play a crucial role. Among the treatment options in physiotherapy, dry needling may offer multiple benefits for athletes but its effects on the various variables that constitute vertical jump performance are minimal. Therefore, the hypothesis of our study is that DN applied to LMTP has positive effects on vertical jump performance. This study aimed to assess the impact of DN on LMTP on the vertical jump performance in female volleyball players.

## 2 Material and methods

### 2.1 Study design

This study employed a randomized clinical trial design, conducted the Department of Invasive Physiotherapy of the European University of Madrid, following the Consolidated Standards of Reporting Trials (CONSORT) guidelines (21). The current study was approved by the Research Ethics Committee (King Juan Carlos University, registration number 0610202334623). The study design was registered at ClinicalTrials.gov (NCT06184672). This study respected the 1964 Helsinki guidelines throughout the study. In addition, all the participants read and signed the informed consent form before being part of this study. This study was conducted between February and March 2024.

### 2.2 Participants

For the recruitment of subjects, informational meetings were held with various professional women's volleyball teams, where the characteristics and procedures of the study were explained. The sample collection took place in February 2024. Initially, 40 female volleyball players were recruited. The inclusion criteria were as follows: healthy female volleyball practitioners with no lower limb injuries in the last 6 months, the presence of LMTP in the gastrocnemius, and familiarity with performing Counter Movement Jump (CMJ) tests. Exclusion criteria comprised needle phobia, active trigger points in the lower limb, any pathology preventing the use of DN, and any condition hindering CMJ test performance.

With patients in prone position, latent trigger points in the gastrocnemius muscles of both legs were identified according to the following criteria: (1) palpable taut band, (2) presence of pain upon palpation of the taut band, (3) local twitch response (LTR) upon palpation of the taut band (22). Palpatory diagnosis has proven to be a validated and reliable assessment technique for identifying the presence of LMTP (23, 24). Once the LMTP was diagnosed, two marks are made on the skin with a dermal pencil, one parallel to the muscle fibers and another perpendicular to them, leaving the LMTP between them. The location of the LMTP was reflected separately on an anatomical sheet of the gastrocnemius muscles.

This study was performed by physiotherapists with more than 6 years of experience and 30 h of weekly clinical practice in order to achieve good inter-examiner reproducibility in the diagnosis of LMTP (25).

### 2.3 Randomization and blinding

Subjects meeting these criteria were randomly assigned to two groups: experimental group (EG) and control group (CG). Opaque envelopes containing numbered cards (1–26) were sealed, and participants randomly selected a card. Odd-numbered subjects constituted the control group, while even-numbered subjects comprised experimental group. This process was carried out by a third party who did not participate in the study. Both the evaluator and the data analyst were blinded to which group each participant belonged.

### 2.4 Sample size

The sample size was determined using G*Power Software (3.1.9.2), based on vertical jump heigth values data obtained on other previous study in which significant improvements in vertical jump height were obtained through the application of deep dry needling in the gastrocnemius muscle, which plays an important role in force and power transfer during the jump by coordinating this transfer from the proximal joints to the ankle (26) with an alpha error of 0.05, a beta error of 0.2, and a medium effect size (*f* = 0.25 or Eta partial squared = 0.06), estimating a sample size of 20 participants. 30% estimated dropout rate was considered due to the study design. Therefore, a total sample size of 26 participants, divided into two groups (*n* = 13), was determined.

### 2.5 Intervention

**Figure 1:**
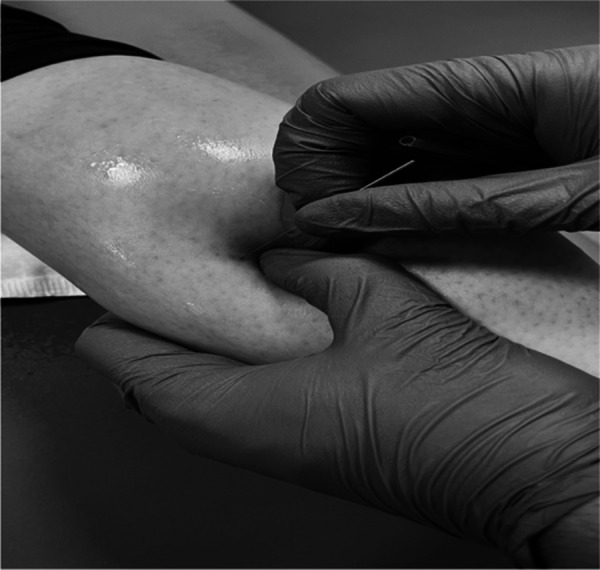
Deep dry needling intervention in gastrocnemius LMTP.

The area was disinfected by the use of 0.5% chlorhexidine digluconate alcohol solution. The physiotherapist in charge of the intervention was specialized on DN therapy and had more than 15 years’ experience with this method for LMTP treatment. In this study, the fast-in and fast-out procedure described by Hong (6) using DN needles with dimensions of 0.30 × 50 mm was utilized. This technique, designed by Hong, applies speed both at the entrance, in order to provoke the local spasm response and at the exit, so that the needle is not inside the taut band once the contraction occurs and is placed in the subcutaneous tissue. The inputs and outputs were performed until the LTR disappears or until the tolerance threshold of the patient is reached (Figure 1).

Immediately after performing the DN technique, ischemic compression was performed on the treated muscle, in order to decrease the duration and intensity of post-puncture pain. For this, a digital pressure was made to the point where the patient's sensation ceases to be pressure and becomes pain (27).

The control group did not receive any intervention. The patients only waited seated the same amount of time that the intervention and the activation group needed to finish their protocol (around 10 min).

### 2.6 Outcomes

The initial evaluation was conducted in both groups after subjects completed a standard warm-up session involving continuous running, dynamic lower limb stretching, and vertical jumps for 10 min (T1). Subsequently, the principal investigator applied the DN technique to the experimental group participants. The second measurement was taken immediately after the needling technique (T2). Subjects in the control group waited on the intervention stretcher for a comparable duration to those who received treatment. The third, fourth and fifth assessments were performed in the same clinical setting, after the same warm-up session and approximately at the same time of day after 24 h (T3), 72 h (T4) and 7 days (T5), respectively. The same five measurements of the variables (T1–T5) were performed for both groups.

It is important to highlight that both groups carried out the same training protocols during the duration of the study within the periodization of their sports discipline.

#### 2.6.1 Counter movement jump test

The CMJ test involved starting from a standing position with hands placed on the hips, followed by a rapid upward jump achieved by flexing and extending the knees. This plyometric action followed the sequence of “eccentric - isometric - concentric” movements. The CMJ measurements were taken using the force platform Quattro Jump (Kistler; Winterthur, Switzerland). Power (P), velocity (V), flight time (FT), strength (S) and height (H) of the CMJ were assessed in watts (W), meters per second (m/s), milliseconds (ms), Newtons (N) and centimeters (cm) respectively. Each subject performed two trials of the CMJ, with a 10-seconds rest period between jumps and the one with the best values was used for analysis (28).

### 2.7 Statistical analysis

The IBM SPSS Statistics v.22.0 program was used for statistical analysis. The normality of each variable was easily evaluated with the Kolmogorov-Smirnov test. Descriptive statistics were used to examine sample demographics, and measurements were presented as mean ± SD. A 2-way repeated measures ANOVA was used for the outcome variables, investigating the interaction between (experimental group and control group) and the time of evaluation (baseline, post-treatment, 24 h follow-up, 72 h follow-up, 1week follow-up). The significance level was predetermined at *p* < 0.05. When differences were identified, Bonferroni's *post hoc* multiple comparisons test was applied. The effect size (ES) was interpreted using Cohen's scale (29): low (<0.20), medium (0.50) and high (>0.80).

## 3 Results

### 3.1 Demographic data

**Figure 2:**
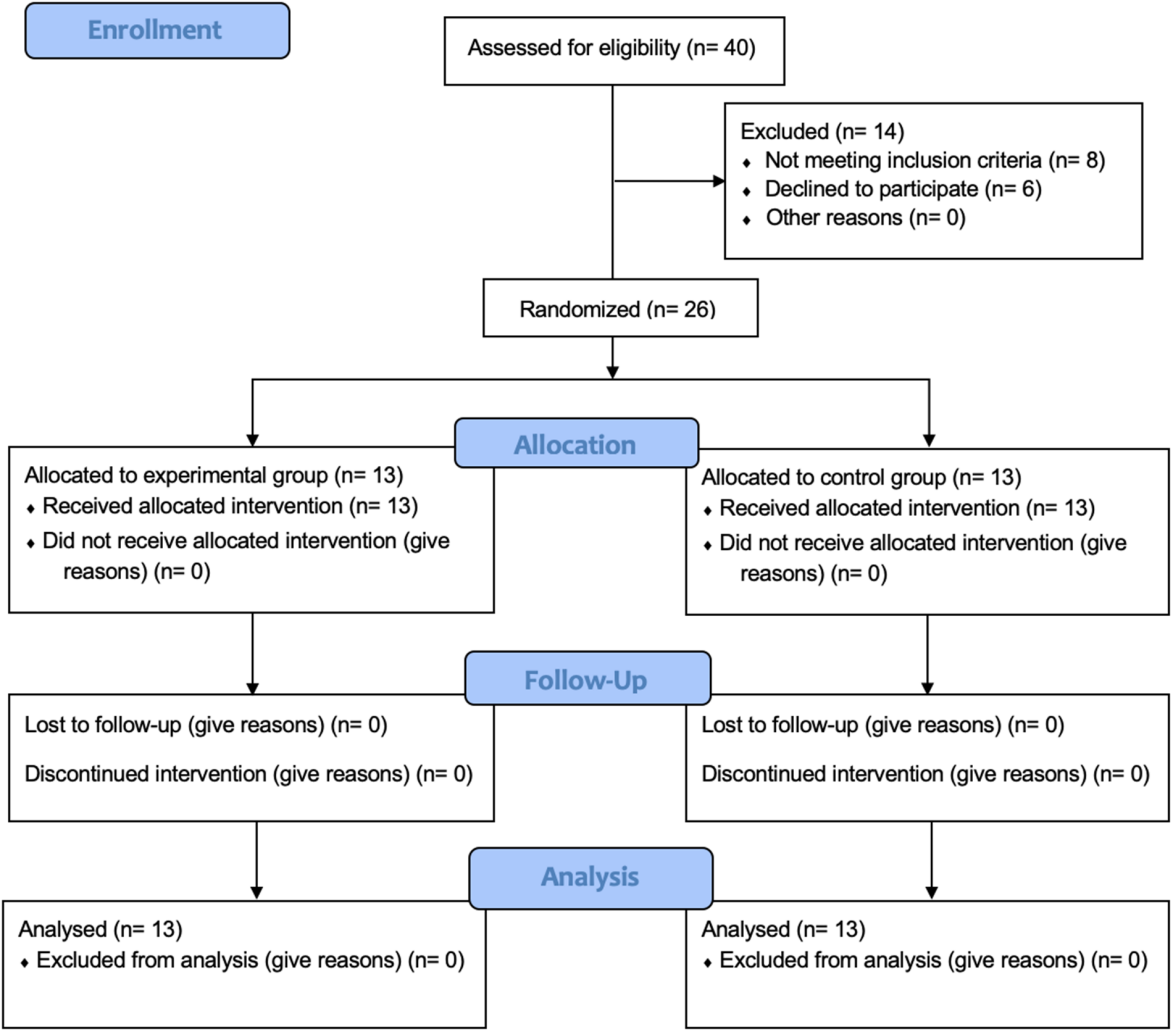
Flow diagram of the study.

Finally, twenty-six healthy female professional Spanish volleyball players with a training frequency of 4–5 days per week who met the inclusion criteria and did not exhibit any exclusion criteria were recruited. All of them consented to participate in the study. They were distributed into the Experimental group (13 women), and the Control group (13 women). The CONSORT flowchart is included (Figure 2). There were no dropouts due to adverse effects of the therapies performed, nor complications after the intervention or during follow-up. No significant differences were found between the experimental group and the control group in demographic characteristics (Table 1).

**Table 1:** Demographic and clinical characteristics of subject.

	EG (*n* = 13)	CG (*n* = 13)	*p*
Sex (male/female)	0/13	0/13	
Age (years)	21.00 ± 2.04	21.08 ± 2.06	n.s
Weight (kg)	67.92 ± 7.78	66.15 ± 7.36	n.s
Height (cm)	176.08 ± 8.93	174.31 ± 0.8.84	n.s

EG, experimental group; CG, control group.

### 3.2 Changes in CMJ after dry needling intervention

The results of the study revealed notable changes in vertical jump variables such as vertical jump height, flight time, velocity, strength, and power. Specific data for these parameters can be found in Table 2, while the mean differences between the groups are presented in Table 3.

**Table 2:** Outcome measurements of counter movement jump variables.

	Baseline	Posttreatment	24 h follow-up	72 h follow-up	1 week follow-up		*f*	*p*	*n* ^2^	pot
Vertical jump (cm**)**										
EG	26.70 ± 5.17	23.24 ± 4.24**	27.79 ± 5.46**	29.25 ± 5.36**	32.93 ± 4.04**^,^^##^	Group	0.26	0.62	0.01	0.08
CG	26.83 ± 5.75	26.89 ± 5.94	26.64 ± 5.47	27.15 ± 5.67	27.17 ± 5.82	Time	91.43	<0.01	0.95	1
						Group × Time	74.94	<0.01	0.94	1
Flight time (ms)										
EG	466.54 ± 48.14	426.73 ± 54.63*	478.38 ± 44.07**	489.31 ± 40.54**	517.15 ± 33.04**^,^^#^	Group	0.18	0.68	0.01	0.07
CG	466.62 ± 49.22	466.69 ± 51.98	465.23 ± 45.82	469.77 ± 47.26	471.92 ± 50.35	Time	32.42	<0.01	0.86	1
						Group × Time	23.44	<0.01	0.82	1
Velocity (m/s)										
EG	1.14 ± 0.12	1.10 ± 0.12**	1.17 ± 0.11*	1.20 ± 0.10**	1.27 ± 0.08**^,^^#^	Group	0.09	0.77	<0.01	0.06
CG	1.15 ± 0.12	1.14 ± 0.13	1.14 ± 0.11	1.16 ± 0.10	1.16 ± 0.12	Time	37.42	<0.01	0.88	1
						Group × Time	26.89	<0.01	0.84	1
Strength (N)										
EG	1,697.45 ± 419.40	1,527.51 ± 383.93**	1,753.35 ± 447.54*	1,816.63 ± 446.72**	1,983.60 ± 468.57**	Group	0.42	0.52	0.02	0.10
CG	1,648.90 ± 251.67	1,658.57 ± 267.18	1,645.07 ± 254.46	1,692.14 ± 275.19	1,684.41 ± 246.82	Time	21.82	<0.01	0.81	1
						Group × Time	16.51	<0.01	0.76	1
Power (W)										
EG	1,942.99 ± 558.10	1,690.54 ± 492.88**	2,031.55 ± 598.23*	2,136.56 ± 600.66**	2,371.14 ± 637.58**^,^^#^	Group	0.48	0.49	0.02	0.10
CG	1,906.82 ± 379.90	1,907.24 ± 403.20	1,886.94 ± 365.39	1,953.49 ± 395.32	1,867.01 ± 370.30	Time	33.27	<0.01	0.86	1
						Group × Time	18.17	<0.01	0.78	1

EG, experimental group; CG, control group.

Values are mean ± SD.

* *P* < 0.05.

** *P* < 0.01, posttreatment, 24-h follow-up, 72-h follow-up and 1week follow-up compared with baseline.

^#^ *P* < 0.05.

^##^ *P* < 0.01, comparisons between the EG and CG at corresponding time points.

**Table 3:** Mean differences between groups from baseline to one-week follow-up on counter movement jump variables.

	EG	CG	Difference	*p*	Effect size
Vertical jump (cm**)**					
Difference	6.22 ± 1.67	0.34 ± 0.91	5.88 (4.80–6.97)	<0.01	3.52
Flight time (ms)					
Difference	50.62 ± 19.60	5.31 ± 9.97	45.31 (32.72–57.90)	<0.01	4.54
Velocity (m/s)					
Difference	0.13 ± 0.05	0.02 ± 0.04	0.11 (0.08–0.45)	<0.01	2.75
Strength (N)					
Difference	286.15 ± 143.26	35.51 ± 66.78	250.64 (160.16–341.11)	<0.01	3.75
Power (W)					
Difference	428.16 ± 170.42	−39.81 ± 299.93	467.96 (267.50–665.43)	<0.01	1.56

EG, experimental group; CG, control group.

Values are mean ± SD.

In the analysis of vertical jump height, the experimental group showed a significant improvement during the 1-week follow-up compared to the control group (*p* < 0.01). Initially, there was a decrease in jump height post-treatment, with a reduction of −3.47 ± 1.69 cm (*p* < 0.01) observed within the experimental group. However, by the 1-week follow-up, this group experienced a notable rebound, with an increase of 6.22 ± 1.67 cm (*p* < 0.01).

A similar pattern emerged in the analysis of flight time. The experimental group outperformed the control group at the 1-week follow-up (*p* < 0.05). Initially, flight time within the experimental group decreased by −39.81 ± 20.07 ms after treatment (*p* < 0.01), but then it significantly increased by 50.62 ± 19.60 ms at the 1-week follow-up (*p* < 0.01).

When examining jump velocity, the experimental group again demonstrated superior results at the 1-week follow-up compared to the control group (*p* < 0.05). After treatment, velocity within the experimental group initially declined by −0.09 ± 0.04 m/s (*p* < 0.01). Yet, during the follow-up period, there was a significant improvement, with velocity increasing by 0.13 ± 0.05 m/s (*p* < 0.01).

In terms of jump strength, no significant differences were observed between the experimental and control groups at any point (*p* > 0.05). Nonetheless, within the experimental group, there was a post-treatment decrease in strength by −169.94 ± 108.37 N (*p* < 0.01), which was followed by a substantial increase of 286.15 ± 143.26 N at the 1-week follow-up (*p* < 0.01).

Finally, in the jump power analysis, the experimental group exhibited significantly higher power at the 1-week follow-up compared to the control group (*p* < 0.05). Initially, power within the experimental group decreased post-treatment by −252.45 ± 123.34 W (*p* < 0.01), but this was followed by a significant increase of 428.16 ± 170.42 W during the follow-up period (*p* < 0.01).

## 4 Discussion

The results obtained in this study seem to suggest a reduction in vertical jump performance after treatment, as well as an improvement in performance after 24 h, reaching maximum values one-week after treatment of LMTP in the gastrocnemius muscles using DN.

Vertical jump constitutes a fundamental component in volleyball performance and is involved in fundamental aspects of the game such as defensive and offensive actions (19), where most actions include accelerations, decelerations and multidirectional movements (30). Furthermore, vertical jump performance can be affected by both muscular and neural components and is closely related to the professional category in which athletes participate, with those with higher vertical jump values being those who play in higher divisions (20).

Previous research (5, 31) reported a high prevalence of LMTP in the lower limbs of asymptomatic individuals, with the gastrocnemius muscles being most frequently affected. The prevalence appears to be higher in women than in men (5) which may be due to genetic, hormonal, social, and even cultural factors (32). Notably, the most common diagnostic finding for LMTP was the presence of a palpable tense band, the primary diagnostic criterion used in this study. Hong DN technique was employed for treatment, recognized as one of the most effective methods (2, 6). Numerous studies have demonstrated the clinical efficacy of this technique in reducing pain (33, 34), improving joint range of motion (27, 34) and even enhancing muscle strength (35).

In accordance with our findings, an immediate decrease in vertical jump height occurred after the intervention in the experimental group. These results are expected due, among other factors, to the neuromuscular damage induced by the rapid needle manipulation (36), producing post-needling soreness that may influence muscle properties, potentially lasting up to 24 h, and completely disappearing 72 h after treatment (37). Furthermore, previous studies have demonstrated a lower contraction velocity 30 min after DN treatment of MLTP (38). On the other hand, at 72 h post-intervention, clinical improvements were observed within the experimental group, although they did not reach statistical significance between groups. Earlier studies employing the same LMTP technique similarly reported improvements at 72 h in variables such as muscle fatigue (39), muscle contraction velocity and muscle stiffness (38). Results obtained at one-week post-intervention reveal significant improvements in the experimental group compared to the control. Among the possible mechanisms by which these benefits are produced through dry needling treatment are the restoration of resting length due to the effect of the local twitch response on actin and myosin, the activation of descending pain inhibitory systems producing an analgesic effect which may reduce nociceptive stimulation, and hemodynamic improvements that facilitate the muscle recovery process (40, 41). Apart from the improvements mentioned in the previously cited literature, other studies have shown that regeneration of the microlesion induced by deep DN occurs specifically after seven days (42).

Various studies published in the current literature have investigated the effects of deep DN in LMTP in sports. Diverse studies conducted across various sports disciplines demonstrate that DN treatment appears to be an effective method for improving recovery and sports performance, especially in situations such as delayed onset muscle soreness or muscle fatigue (43, 44). In triathletes, the treatment with deep DN on the LMTP have demonstrated improvements in electromyographic activity of the gastrocnemius muscles (45), as well as a decrease in pressure pain threshold (46). Haser et al. (47) investigated the effects of deep DN on trigger points of hip flexors and extensors, resulting in an improvement in muscular endurance and range of motion in hip flexors, as well as an improvement in maximum strength of hip extensors after treatment in elite soccer players. In addition to the effects produced on muscle strength, the presence of LMTP can affect the fatigue and control of muscle activity (48), and it has been shown that its treatment by deep DN produced an improvement in neuromuscular control and static balance in basketball players (49). Furthermore, a recent study demonstrated that deep DN in LMTP improved blood perfusion and biomechanical properties of the gastrocnemius in professional mixed martial arts athletes (50). Regarding the influence of DN on vertical jump, our study aligns with the findings of Devereaux et al. concerning the immediate decrease in jump capacity, with improvement in these variables observed after 48–72 h (26). Additionally, deep DN in sports shows minor adverse effects, safety, and improvements in variables such as flexibility, strength, power, and pain (51).

### 4.1 Limitations and future research

While this study provides valuable insights, several limitations exist. Future studies should explore the timing of DN interventions concerning competition schedules.

Besides, nowadays there is lack of clinical consensus about certain variables regarding DN, such as needle insertion velocity, depth and number of twitch responses, which may infer a wider variability of outcomes. Additionally, the use of more objective tools for diagnosing LMTP, such as pressure algometry, could have provided greater objectivity to the study.

On the one hand, the presence of LMTP has not been evaluated in other muscle groups of the lower limb involved in the vertical jump, such as quadriceps. On the other hand, having the control group undergo an alternative intervention such as stretching or muscle activation could have enhanced the validity of our results.

Additionally, the study prompts further investigation into DN as a therapeutic tool for injury prevention, considering its potential impact on lower limb strength.

### 4.2 Practical applications

The results of this study, although they must be interpreted with caution, may have various practical applications focused on improving recovery and athletic performance. On one hand, the treatment of latent trigger points in the gastrocnemius muscle, aimed at promoting recovery or enhancing performance, should not be performed prior to training or competition if there is not a minimum of 24 h of rest between the intervention and sports practice, due to the observed decrease in performance. On the other hand, to achieve optimal benefits on vertical jump performance in female volleyball players, the intervention should be carried out at least one week before the competition.

## 5 Conclusion

Deep DN of the LMTP in the gastrocnemius muscles immediately decreases vertical jump performance and improves it after 24 h post-intervention in healthy female volleyball players, with significant improvements observed one week after the intervention.

## Data Availability

The raw data supporting the conclusions of this article will be made available by the authors, without undue reservation.
